# *Legionella* Colonization of Hotel Water Systems in Touristic Places of Greece: Association with System Characteristics and Physicochemical Parameters

**DOI:** 10.3390/ijerph15122707

**Published:** 2018-11-30

**Authors:** Maria A. Kyritsi, Varvara A. Mouchtouri, Antonis Katsioulis, Elina Kostara, Vasileios Nakoulas, Marina Hatzinikou, Christos Hadjichristodoulou

**Affiliations:** 1 Department of Hygiene and Epidemiology, Medical School, University of Thessaly, 41222 Larissa, Greece; mkiritsi@med.uth.gr (M.A.K.); mouchtourib@med.uth.gr (V.A.M.); akatsioul@med.uth.gr (A.K.); elkost@med.uth.gr (E.K.); nakulasb@yahoo.gr (V.N.); marinahatzinikou@yahoo.gr (M.H.); 2 Regional Public Health Laboratory of Thessaly, 41222 Larissa, Greece

**Keywords:** hotel-associated, Legionnaires’ disease, Greece, physicochemical, cold water

## Abstract

This study aimed to assess the colonization of hotel water systems in central Greece and Corfu by *Legionella*, and to investigate the association between physicochemical parameters and *Legionella* colonization. Standardized hygiene inspection was conducted in 51 hotels, and 556 water samples were analyzed for *Legionella* spp. Free chlorine concentration, pH, hardness, conductivity, and trace metals were defined in cold water samples. The results of inspections and chemical analyses were associated with the microbiological results using univariate and logistic regression analysis. According to the score of the checklist used for the inspections, 17.6% of the hotels were classified as satisfactory, 15.7% as adequate, and 66.7% as unsatisfactory. Moreover, 74.5% of the hotels were colonized by *Legionella* spp. and 31.4% required remedial measures according to the European guidelines. *Legionella* spp. were isolated in 28% of the samples. Unsatisfactory results of inspections were associated with *Legionella* presence (relative risk (RR) = 7.67, *p*-value = 0.043). In hot-water systems, <50 °C temperatures increased the risk of *Legionella* colonization (RR = 5.36, *p*-value < 0.001). In cold-water systems, free chlorine concentration <0.375 mg/L (odds ratio (OR) = 9.76, *p*-value = 0.001), pH ≥ 7.45 (OR = 4.05, *p*-value = 0.007), and hardness ≥321 mgCaCO_3_/L (OR = 5.63, *p*-value = 0.003) increased the risk, whereas copper pipes demonstrated a protective role (OR = 0.29, *p*-value = 0.0024). The majority of the hotels inspected were colonized with *Legionella*. Supplementary monitoring of the risk factors that were identified should be considered.

## 1. Introduction

*Legionella* spp. are Gram-negative bacteria causing Legionnaires’ disease (a severe pneumonia with potential multisystem offence and an often fatal outcome), and Pontiac fever (a mild flu-like febrile syndrome without clinical manifestations of pneumonia in humans) [1]. The elderly, as well as immunosuppressed individuals, are more prone to *Legionella* infection [2]. Currently, at least 60 *Legionella* species and more than 70 serogroups of the microorganism have been identified [3]. *Legionella* spp. is a common inhabitant of both natural and artificial aquatic environments [4].

Legionnaires’ disease constitutes a significant public health issue worldwide. The majority of cases are sporadic and community-acquired (70%), followed by travel-associated (20%), and healthcare-associated (10%). The general case fatality rate of Legionnaires’ disease is high at approximately 10%. For hospital-acquired cases, the case fatality rate reaches and can exceed 25%, while travel-associated cases reach a 6% case fatality rate [5]. As a general rule, the reporting of Legionnaires’ disease is heterogeneous among different countries [6,7]. Concerning Greece, the incidence rate for Legionnaires’ disease was 2.5/million inhabitants, with 74% of the cases community-acquired, 11% healthcare-associated, and 15% travel-associated cases. The overall mortality was 12% [5]. In 2014, the total number of Legionnaires’ disease cases reached 41; eight travel-associated cases were recorded in 2013 and four in 2014 [8].

In 2013, Greece had a total of 9,667 hotels, which included a total of 401,332 rooms [9,10]. Oftentimes, more than one hotel is implicated in reported cases of Legionnaires’ disease. Indeed, in 2011, 14 cases of Legionnaires’ disease were confirmed in residents from England and Wales with a history of travel to Corfu, Greece. Epidemiological investigations and microbiological analysis of clinical and environmental samples excluded a single source and implicated several accommodation sites as sources of sporadic infection [11].

The main reason for legionellosis in travelers can be attributed to the poor maintenance of hotel plumbing systems. The absence of regular cleaning and disinfection results in salt accumulation and biofilm formation and the subsequent colonization of *Legionella*, one of the most frequent members of the water distribution system (WDS) microbiota [12,13]. High environmental temperatures and the periodic operation of water plumbing systems (that create both warm and stagnant water) induce the proliferation of aquatic opportunistic pathogens such as *Legionella* [12,13]. In Greece, both factors are present in hotel water supply systems [14]. Studies regarding hotel colonization with *Legionella* were conducted in Greece from Alexiou et al. in 1989, Mouchtouri et al. in 2007, and finally, Fragou et al. in 2012; however, none of the aforementioned studies evaluated the influence of physical and chemical characteristics of water on *Legionella* presence. Similarly, the majority of the published data regarding hotel colonization with *Legionella* focus on hot-water systems, since these temperatures favor *Legionella* growth [15]. A recent review that evaluated 136 Legionnaires’ disease and Pontiac fever outbreaks between 2006 and 2017 highlighted the importance of building water systems as major contributors to outbreak-related cases and deaths [16].

In Greece, especially during the summer months, the water pipes in hotels are exposed to high temperatures, and the temperature of circulating water often reaches the optimum temperature for *Legionella* growth. For this reason, the evaluation of cold-water systems is equally important. This study was designed in order to explore and assess associations between specific physicochemical parameters of cold-water distribution systems (WDS) and *Legionella* colonization. The overall aim was to identify potential risk factors and promote the implementation of preventive measures.

## 2. Materials and Methods

Hygiene inspections and water sampling were conducted in 51 hotels in central Greece and Corfu. Corfu was chosen because of the increase in travel-associated cases of Legionnaires’ disease as recorded by the European Legionnaires’ Disease Surveillance Network (ELDSNet) in 2011 [8]. In the region of Thessaly, the inspections were performed after the Hellenic Center for Disease Control and Prevention (HCDCP) recommended routine control to touristic accommodations. 

Overall, 556 water samples were collected between October 2011 and December 2012. In detail 496 samples were collected from showers, 36 samples from swimming pools, eight samples from taps, two samples from coolers, three samples from boilers, three samples from cold-water tanks, four samples from hot tubs, one sample from a fountain, and three water samples from cooling towers. The sampling was performed in accordance with International Organization for Standardization (ISO) 5667-5 [17]. The samples that were intended for microbiological analysis were collected in sterilized 500-mL glass bottles containing a sufficient amount of sodium thiosusulfate (Na_2_S_2_O_3_ × 5H_2_O), in order to neutralize the disinfectant action. The samples intended for physicochemical analysis were collected in sterilized 500-mL glass bottles. The sampling was representative of the water distribution system of each hotel, and included cold- and hot-water supply systems, swimming pools, and hot tubs (spas). For each sample an appropriate sampling form was filled out which denoted the date and time of the sampling, the hotel name, the sampling point, the disinfectant used, the free disinfectant concentration, the temperature of the water, and the pH measured. Cold-water samples were transferred to the laboratory under refrigeration at a maximum temperature of 6 °C, and hot-water samples at room temperature within 48 h after sampling.

**Figure 1:**
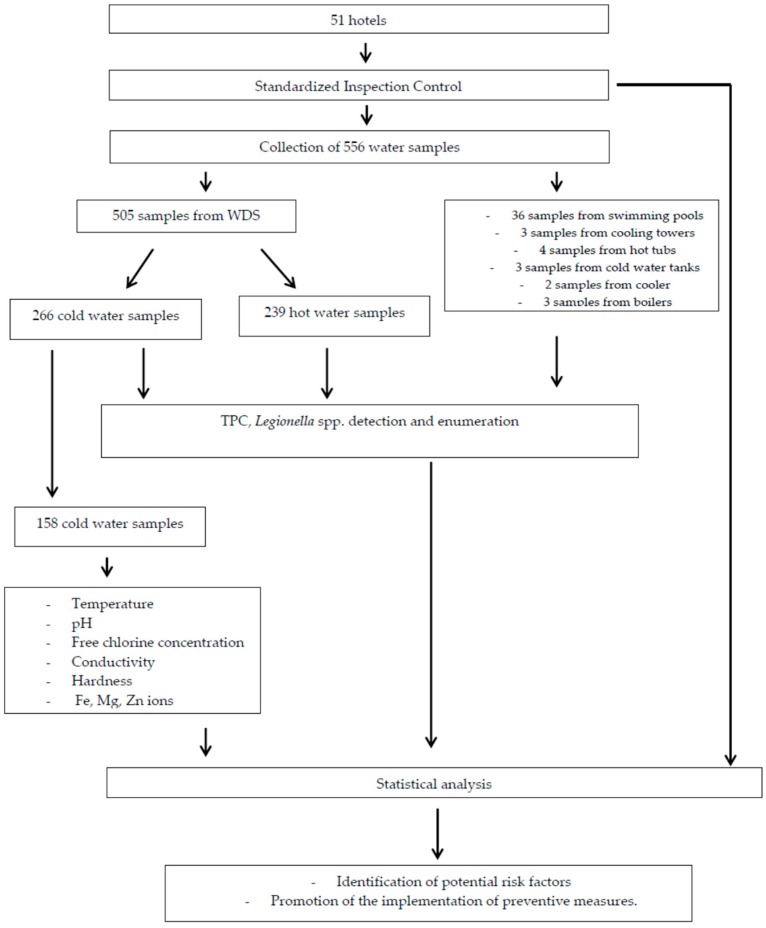
Flowchart of the methodology used for the study. WDS: Water Distribution System; TPC: Total Plate Count.

The workflow of the methodology is demonstrated in Figure 1.

### 2.1. Hygiene Inspection and Risk Assessment

Hygiene inspections of each touristic accommodation were conducted in order to estimate the risk of *Legionella* colonization using a checklist as described by Hadjichristodoulou et al. [18]. The items included in the reports were based on requirements of national and European legislation and World Health Organization guidelines. The inspection included “critical” and “non-critical” items. Critical items were violations “which are more likely to contribute to water contamination, illness, or environmental degradation and represent substantial public health hazards and most likely associated with potential waterborne disease transmission” [19].

In the checklist, information concerning water tanks, pipe material, general information about the pressure, the filters, tank decontamination, cold- and hot-water distribution systems, boilers, water storage tanks, taps, and firefighting installations were recorded. Additional information included in the checkbook data of the accommodation, as well as information concerning measurements performed by the health personnel, were recorded (if available). A total of 39 checkpoints were evaluated. Each negative answer received a negative score, with the classification of the hotels as follows: satisfactory operation, total negative rating −7 and no critical check points were observed; adequate operation, total negative rating from −8 to −14; unsatisfactory operation, total negative rating less than −15. The checklist used in order to evaluate the hotels is included in the Appendix A.

### 2.2. Microbiological Analysis

For all the samples collected, the total plate count was defined according to ISO 6222:1999 [20]. One milliliter of the sample, and 10^−1^ and 10^−2^ dilutions were aseptically transferred in sterilized petri dishes of 92 mm. Melted medium plate count agar (LabM, Plate Count Agar) at 45–50 °C were poured into the dishes. After solidification of the medium, the dishes were incubated at an ambient air temperature of 36 ± 2 °C for 44 ± 4 h; after the incubation period, all colonies from all dilutions were counted and the number of colony-forming units (CFU per mL was calculated.

For all the samples collected, *Legionella* detection and enumeration was performed following ISO 11731:1998 indications [21]. Briefly, 500 mL of water was filtered using a 47-mm nitrocellulose membrane with 0.22-µm pores (Millipore, Burlington, MA, USA, Merck, Kenilworth, NJ, USA). After filtration, the membrane was placed in 5 mL of ¼ Ringers’ solution and was vortexed for at least 2 min. One milliliter of the vortexed solution was 1/10 diluted, 1 mL was acid-treated, and another 1 mL was heat-treated. Subsequently, 100 μL from each solution was inoculated into glycine/vancomycin/polymixin/colimicyn (GVPC) medium dishes (Biomerieux, Marcy-l’Étoile, France), and they were incubated at 36 ± 1 °C for 10 days in increased moisture conditions. At least three colonies characteristic of *Legionella* from each GVPC dish were selected and subcultured onto buffered charcoal yeast extract (BCYE) and BCYE without cysteine (BCYE − cys) media. The dishes were incubated at 36 ± 1 °C for two days, and the colonies which grew on BCYE but failed to grow on BCYE − cys medium were regarded as *Legionella* spp. Both the species of *Legionella* and the serogroup were determined using a latex agglutination test (Oxoid Legionella Latex Test). The sensitivity of the method was 100 cfu/L.

### 2.3. Physicochemical Analysis

In total, 158 cold-water samples were submitted to further physicochemical analysis. The following physicochemical parameters were defined for each sample: temperature, pH, free chlorine concentration, conductivity, hardness, and the content of Fe, Mg, and Zn ions.

Temperature, pH, and free chlorine concentration were measured on site. The temperature of all samples collected was measured using a validated digital thermometer. The pH, as well as the free chlorine concentration, was defined using a spectrometer (Hanna Instruments Ltd, Eden Way, Pages Industrial Park, Leighton Buzzard, Bedfordshire, UK ). Free chlorine concentration was expressed in mg/L.

The conductivity of the samples was defined using an electrochemical method, and the measurements were performed with a calibrated Consort conductivity meter. The measurements were expressed in μS/cm.

Both hardness and calcium concentration were determined using titration methods. Hardness expresses the content of the water in polyvalent cations, mainly calcium (Ca^2+^) and manganese (Mg^2+^). In order to define the hardness, 50 mL of the sample was placed in a conical bottle and 4 mL of NH_3_ solution, along with an indicator, was added. Afterwards, ethylenediaminetetraacetic acid (EDTA) solution was added until the color of the solution turned green. The volume of the EDTA solution needed was titrated and multiplied by 20 in order to calculate the value of the hardness in units of mg/L CaCO_3_. Respectively, to calculate the calcium concentration, 50 mL of each sample were placed in a conical bottle, together with 2 mL of NaOH solution and a Calcon indicator. Again, EDTA solution was added until the color of the solution turned blue. The volume of the EDTA solution needed was titrated and multiplied by 8.015 in order to calculate the value of calcium concentration in units of mg/L Ca units, and by 2.5 in order to calculate the value of calcium concentration in units of mg/L CaCO_3_.

Finally, in order to define the heavy metal concentration in the samples collected, atomic absorption spectroscopy (Perkin Elmer, Waltham, MA, USA) was used. Samples were filtered using a 47-mm nitrocellulose membrane with 0.45-µm pores (Millipore, Merck), and acidification of the samples was performed until reaching a final concentration of 0.2% HNO_3_ (0.2 mL HNO_3_ in 100 mL of each sample). Subsequently, each sample was absorbed and inserted as a cloud, either in a flame or in a graphite oven, where the solver is evaporated and the metals’ ions are atomized. When the atoms of the metal pass through their constant intensity beam and wave length, part of the radiation is absorbed and the reduction of the intensity is measured, with the proportionate concentration of the metal ion recorded in μg/L.

### 2.4. Statistical Analysis

All data collected from the study hotels (questionnaire and laboratory results) were entered into an Excel file (Microsoft Office 2013, Microsoft Excel^®^). Statistical analysis was performed using IBM SPSS Statistics software (v.22.0. IBM Corp., Armonk, NY, USA).

Quantitative variables are presented either as mean values with standard deviation or as a median value with the interquartile range (IQR). Qualitative variables are presented as frequencies with percentages. The receiver operating characteristic (ROC) curve analysis was conducted to determine the optimal cut-off values of the physicochemical parameters for *Legionella* colonization.

In the univariate analysis, chi-square test or Fisher’s exact test was used to identify any association between categorical factors (free disinfectant concentration, pH, total plate count (TPC), conductivity, hardness, calcium, and pipe material) and *Legionella* colonization, calculating the relative risks (RRs) and the corresponding 95% confidence intervals (95% CIs). In cases where the relative risk could not be calculated due to zero frequencies, the Haldane correction was applied. In addition, Spearman’s correlation coefficient was calculated to explore any correlation between physicochemical parameters and *Legionella* concentration.

In the multivariate analysis, multiple logistic regression analysis was performed using the backward conditional method to identify the independent risk factors for *Legionella* colonization by calculating the odds ratios (ORs) and the corresponding 95% CIs. *Legionella* presence (≥100 cfu/L) was defined as the dependent variable, and the risk factors that were found to be statistically significant in the univariate analysis were defined as independent variables.

A result with a *p*-value of less than 0.05 was considered to be statistically significant.

## 3. Results

A total of 51 hotels were evaluated and 556 water samples were collected. Thirty-eight hotels (75%) were found to be colonized with *Legionella* spp., while 16 hotels (31.4%) required intervention measures according to the European Working Group for Legionella Infections (EWGLI) criteria. While nine hotels (17.6%) were classified as “satisfactory operation” by the hygiene inspections and eight hotels (15.7%) were classified as “adequate operation”, 34 hotels (66.7%) were classified as “unsatisfactory operation”. The most frequent types of critical points recorded during inspection control included the following: no disinfection of the system occurred when it was not used for over a month; there was presence of stagnant water in the pipes for over a week; the flushing procedure was not applied in the case of stagnant water; there was presence of salts on the showers; regular water sampling was not performed at least every six months; the outgoing cold-water temperature was higher than 25 °C; and residual chlorine concentration was <0.2 mg/L. None of the hotels that were classified with satisfactory operation required corrective actions, whereas, in 16 out of 42 hotels (38.1%) that were classified with adequate or unsatisfactory operation, corrective measures were necessary. Specifically, an unsatisfactory result of the inspection was associated with *Legionella* colonization and the need for corrective intervention (RR = 7.67, *p*-value = 0.043) (Table 1, Appendix A).

**Figure 2:**
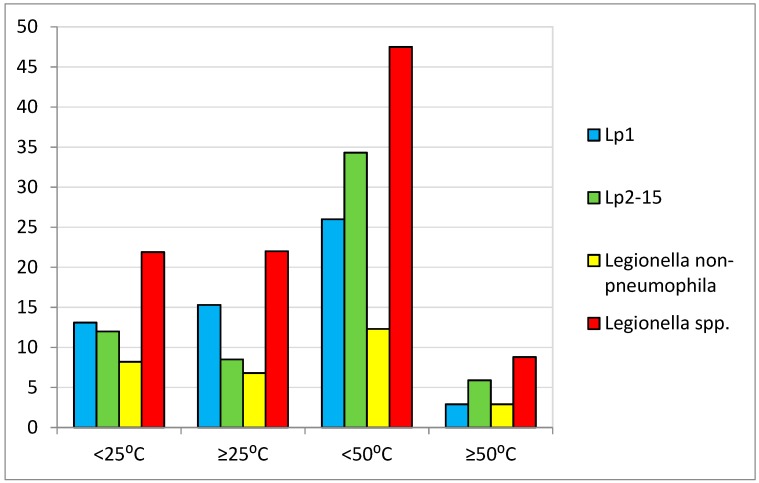
Samples positive for *Legionella* detection in temperature ranges.

*Legionella* spp. were detected (≥100 CFU/L) in 160 out of 556 (28%) of the water samples analyzed. Specifically, 151 samples from showers, five from taps, one from a boiler, and three from hot tubs were positive for *Legionella* detection. The microorganism was detected in 100 out of 239 (41.8%) hot-water samples, in 57 out of 266 (21.4%) cold-water samples, and in three out of four (75%) hot-tub samples. The results of *Legionella* detection and the correlation with water temperature are presented in Table 2, and all positive samples for *Legionella* detection in temperature ranges are demonstrated in Figure 2.

Concerning Fe and Mg ion concentrations, higher values were recorded in positive cold-water samples for *Legionella* spp. detection, but the results were not statistically significant. Zn ion concentrations were similar in both positive and negative samples, as shown in Table 3, where the correlation of all the physicochemical parameters are analyzed, and the colonization of the system with *Legionella* spp. is also demonstrated.

Table 4 demonstrates in detail the association between the statistically significant physicochemical characteristics examined and *Legionella* spp. colonization of the cold-water systems, using univariate analysis. The optimal cut-off values of the physicochemical parameters which favor the colonization of the cold-water system with *Legionella* spp. were defined using the ROC curves. A positive association was found in samples with free chlorine concentration <0.375 mg/L, hardness ≥321 mgCaCO_3_/L, calcium concentration ≥150 mgCaCO_3_/L, TPC ≥2.5 × 10^4^ cfu/mL, conductivity values ≥1775 μS/cm (25 °C), and finally, pH values ≥7.45. There was a negative association with colonization of *Legionella* spp. when the pipes of the hotel plumbing systems were made from copper.

The results from the univariate analysis mentioned above were confirmed by the multivariate analysis (Table 5). Specifically, free disinfectant concentration <0.375 mg/L was found to increase the risk of colonization of cold-water supply systems with *Legionella* spp. regardless of other risk factors (OR = 9.76, 95% CIs: 2.46–38.66). Similarly, pH values ≥7.45 (OR = 4.05, 95% CIs: 1.47–11.19), TPC ≥2.5 × 10^4^ cfu/mL (OR = 2.63, 95% CIs: 0.98–7.09), and hardness concentration ≥321 mgCaCO_3_/L (OR = 5.63, 95% CIs: 1.82–17.41) increased the risk of colonization of cold-water supply systems with *Legionella*. Finally, it is indicated that copper pipes demonstrate a protective role against colonization of the plumbing system with *Legionella* (OR = 0.29, 95% CIs: 0.10–0.85).

## 4. Discussion

In the present study, *Legionella* spp. were found to colonize 75% of the hotels evaluated. Previous studies in Greece demonstrated variations in *Legionella* colonization rates, with 86% [22], 21% [14], and 33% [23] of the hotels examined found to be colonized with *Legionella*. Similar variations were recorded in studies conducted within other countries of the European Union. In Croatia, none of the hotels that remain open throughout the year were found to be colonized, whereas 40% of the hotels with periodic operation were colonized with the bacterium [24]. In Italy, 63%, 75%, and 64% of the hotels examined were found to be colonized; in more recent research by Totaro et al., *Legionella* spp. were detected in 26% of the hot-water networks of 121 residential buildings in Pisa during a three-year survey on *Legionella* presence [25,26,27,28]. In Turkey, 59% were colonized [29], whereas, in a different study, 92% were found to be colonized [30]. Finally, in a study from the United Kingdom that included hotels and hospitals, 67% of the facilities evaluated were found to be colonized with *Legionella* spp. [31]. Despite the aforementioned data, it was not until September 2017 that the Greek authorities published a regulation determining 1000 cfu/L as the upper limit of *Legionella* spp. concentration in water systems of hotel and health care facilities [32].

While the majority of studies highlighted above focus on hot-water networks, in our study, 21.4% of the cold-water samples were positive for *Legionella* spp. This implies the presence of risk factors that favor *Legionella* proliferation in cold-water systems as well. One of the main reasons for this could be the high environmental temperatures in the Mediterranean during the summer months, which leads to increases in the temperature of circulating water in pipes, which is optimum for *Legionella* growth. 

In this study, 31.4% of the hotels evaluated needed intervention measures according to the EWGLI criteria. Concerning the hygiene inspection classification, the majority of the hotels’ WDS (66.7%) were classified as “unsatisfactory”. When a hotel was classified as adequate or unsatisfactory operation, it exhibited an almost eight times higher risk of requiring corrective intervention, whereas none of the hotels that were classified with satisfactory operation needed to undertake corrective measures. These results confirm that a satisfactory level of operation, as it is defined by the use of the checklist, could possibly be reassuring about *Legionella* colonization and underlines the importance of continuous record-keeping of water system data by the competent personnel [18,33]. Concerning the physicochemical analysis of the cold-water samples, levels of free chlorine concentration >0.375 mg/L were found to have a protective effect against *Legionella* colonization and confirmed previous published data that supported the importance of maintaining disinfectant residual [34,14]. Regarding copper pipes, our findings are in concordance with studies that support the antimicrobial effect of copper [1,35,36,37]. On the contrary, pH ≥7.45, TPC ≥2.5 × 10^4^ cfu/mL, conductivity ≥1775 μS/cm (25 °C), hardness ≥321 mgCaCO_3_/L, and calcium concentrations ≥150 mgCaCO_3_/L increased the risk of colonization of cold-water supply systems with *Legionella* spp. Concerning the pH levels, prior studies reported that *L. pneumophila* has reduced viability and cultivability at higher pH levels [13,38,39]. Previous data in hot-water systems also supported that higher TPCs, with lower cut-off values, favor *Legionella* growth [40]. Concerning hardness, and subsequently, calcium ion concentration, the published data are contradictory. Positive associations [41], negative associations [42], and no association [25] were reported, while negative associations between conductivity values and *Legionella* colonization were suggested [41]. Although the plumbing systems of hotels and other large buildings are highly complex, Greek legislation regards that the physicochemical monitoring of the municipal WDS is sufficient to ensure the physicochemical water quality within these facilities. Thus, the hotel operators are not obliged to conduct further controls [32]. 

Our findings support that the implementation of control measures and the monitoring of the systems should be continuous. Checking for damage, corrosion, contamination, visible biofilm formation of the system and its parts, continuous monitoring of the water quality, periodic sampling for *Legionella*, and finally, record-keeping of the aforementioned data by well-trained competent personnel is the basis of a water safety plan that every hotel must implement [43,34]. The physicochemical composition of the water can affect the microbiome in the plumbing systems of buildings, leading to the emergence of opportunistic pathogens such as *Legionella*. Thus, consideration of supplementary monitoring of these specific physicochemical parameters, which appear to influence the colonization and proliferation of *Legionella* in hotel water supply systems, could contribute to prevention of travel-associated Legionnaires’ disease.

## 5. Conclusions

Although there is strong evidence that various physicochemical agents of the water influence the colonization of systems with *Legionella,* further studies must be conducted in order to thoroughly comprehend and evaluate the various parameters affecting *Legionella* proliferation as part of the water microbiome. The present study offers a complete assessment of hotel water supply systems combining standardized inspections, risk assessment, physicochemical measurements, and microbiological analyses for *Legionella* of a large number of water samples. We propose consideration of supplementary monitoring of water hardness and evaluation of pipe material, which appear to influence the colonization and proliferation of *Legionella* in hotel WDS, in order to prevent travel-associated Legionnaires’ disease cases. The high case fatality ratio of hospitalized cases and the extensive publicity that travel-associated cases usually receive, in combination with the great importance of tourism not only to Greece but also internationally, should raise the awareness of both public health authorities and the hotel operators, in order to prevent and promptly identify cases.

## Figures and Tables

**Table 1:** Hygiene inspection classification score and need for intervention measures according to European Working Group for Legionella Infections (EWGLI) criteria. RR—relative risk.

Classification	Intervention Measures			
	Frequency	*%*	RR *	*p*-Value **
Adequate or unsatisfactory (score ≤−7)	16 out of 42	38.1	**7.67**	**0.043**
Satisfactory (score 0 to −6)	0 out of 9	0.0		

* Haldane correction. ** Fisher’s exact test.

**Table 2:** Correlation between water temperature and *Legionella* detection. CIs—confidence intervals.

Parameter	Hot Water (*n* = 239)		Cold Water (*n* = 266)				
	Frequency	%	Frequency	%	RR	95% CIs	*p*-Value *
*Legionella* spp.	100	41.8	57	21.4	1.95	1.48–2.57	<0.001
*Lp* sg. 1	54	22.6	35	13.2	1.72	1.16–2.53	0.005
*Lp s.g.* 2–15	72	30.1	30	11.3	2.67	1.81–3.94	<0.001
*Legionella non-pneumophila*	26	10.9	20	7.5	1.45	0.83–2.52	0.190

* Chi-square test. CIs: Confidence intervals; *Lp*: *Legionella pneumophila*; s.g.: serogroup.

**Table 3:** Correlation of water physicochemical parameters and colonization of the system with *Legionella* spp. CFU: colony-forming unit.

*Legionella* spp.			
	Correlation Index (*)	*N* (Number of Samples Analyzed)	*p*-Value
**Free disinfectant concentration (mg/L)**	−0.285	157	**<0.001**
**pH**	**0.188**	157	**0.018**
**Total aerobic count (cfu/mL)**	**0.230**	158	**0.004**
**Conductivity (μS/cm) (250C)**	**0.175**	144	**0.036**
**Hardness (mg CaCO_3_/L)**	**0.222**	144	**0.008**
**Calcium (mg CaCO_3_/L)**	**0.170**	144	**0.041**
**Iron (Fe) (μg/L)**	−0.010	155	0.899
**Zinc (Zn) (μg/L)**	0.116	40	0.477
**Manganese (Mn) (μg/L)**	0.052	139	0.546

(*) Spearman correlation index.

**Table 4:** Univariate analysis for colonization of water supply systems with *Legionella* spp.

Parameter		*Legionella* spp. (+)				
		Frequency	%	RR	95% CIs	*p*-Value *
Free disinfectant concentration (mg/L)	<0.375	33/89	37.1	**8.40**	2.60–26.25	**<0.001**
	≥0.375	3/68	4.4			
pH	≥7.45	23/67	34.3	**2.38**	1.30–4.34	**0.003**
	<7.45	13/90	14.4			
Total aerobic count (cfu/mL)	≥2.5 × 10^4^	24/69	34.8	**2.58**	1.39–4.78	**0.002**
	<2.5 × 10^4^	12/89	13.5			
Conductivity (μS/cm) (25 °C)	≥1775	14/28	50	**2.90**	1.68–5.00.	**<0.001**
	<1775	20/116	17.2			
Hardness (mg CaCO_3_/L)	≥321	28/72	38.9	**4.67**	2.06–10.59	**<0.001**
	<321	6/72	8.3			
Calcium (mg CaCO_3_/L)	≥150	29/92	31.5	**3.28**	1.35–7.95	**0.003**
	<150	5/52	9.6			
Pipe material	Copper (+)	8/66	12.1	**0.40**	0.19–0.82	**0.007**
	Copper (−)	28/92	30.4			

* Chi-square test. RR: Relative Risk.

**Table 5:** Multivariate analysis for colonization of water supply systems with *Legionella* spp.

Parameter		OR	95% CIs	*p*-Value
Free disinfectant concentration (mg/L)	<0.375 vs. >0.375	9.76	2.46–38.66	0.001
pH	≥7.45 vs. <7.45	4.05	1.47–11.19	0.007
Total aerobic count (cfu/mL)	≥2.5 × 10^4^ vs. <2.5 × 10^4^	2.63	0.98–7.09	0.056
Hardness (mg CaCO_3_/L)	≥321 vs. <321	5.63	1.82–17.41	0.003
Pipe material	Copper (+) vs. Copper (−)	0.29	0.10–0.85	0.024

OR: Odds Ratio.

## Appendix A

**Table A1:** Checklist used for standardized hygiene inspection.

No	Checkpoint	YES √	NO ✕	Observations
**General points**				
1	Meter pressure 1–12 atm		−1	
2	Filters in good condition		−2	
3	Insulation in good condition		−2	
4	Absence of leaks in the system		−2	
5 *	The tank is well maintained and there are no regiments		−3	
6	There are covers over the tanks and meshes of wire over every open water pipe		−1	
7	The amount of the stored water is used within one day		−1	
8 *	The system is cleaned and disinfected when it is not used for over a month		−3	
9 *	The system and the tanks are treated with the proper disinfectants at least once in a year		−3	
10	Water supply is not interrupted for a long time		−1	
11	Taps that are not in use are removed from the system		−2	
12	Check on the water system outline			
**Cold-water system**				
13	The coolers are in a good condition		−1	
14	The filters of the coolers are in a good condition		−1	
**Hot-water system**				
15	The system responds sufficiently in rush hours		−1	
16	The is no change in water consumption		−1	
17 *	Absence of stagnant water in the pipes for over a week		−3	
18 *	If NO, flushing procedure is applied		−3	
19 *	The showers are clean without salts		−3	
**Water heaters and water storage devices**				
20	The device is dried and controlled		−1	
21	The device is cleaned if necessary		−2	
22	The hot-water export pipe is drained		−1	
23	They are well maintained		−2	
**Batteries**				
24	Operated and maintained according to the manufacturer’s advice		−2	
**Water fire-fighting facilities**				
25	There is no water regression from the fire-fighting water to the water supply system		−2	
**ANNEX I: Checkbook data**				
26	There is a check book		−2	
27 *	Regular water sampling is performed at least every 6 months		−3	
28	There are no positive results recorded in the checkbook (if there are any)		−2	
29 *	No *Legionella* detection above 10 cfu/10 mL was recorded in the past 6 months		−3	
**ANNEX II: Measurements by the health service personnel**				
30 *	Outgoing cold-water temperature is lower than 25 °C		−3	
31	Tap cold-water temperature is lower than 25 °C, after two minutes of flow		−2	
32	Hot-water temperature is at least 50 °C, after one minute of flow		−2	
33 *	The variation between two serial temperature measurements of hot water with a flow interval of one minute should not exceed 10 °C		−3	
34	The water is stored and distributed at 60 °C		−2	
35	There is no temperature stratification of the water circulating in the heating and storage water devices		−1	
36	If the system is indirect, the temperature of the water coming out from the heating device should be at least 60 °C, and that of the returning water should be at least 50 °C		−2	
37	The pH measured is between 6.5–8.5		−2	
38 *	The residual chlorine measured is between 0.2–0.5 mg/L		−3	
39	Absence of problems in taste or in odor		−1	

Result of the inspection: satisfactory operation (total negative rating up to −7; no critical checkpoints observed); adequate operation (total negative rating ranging from −8 to −14); unsatisfactory operation (total negative rating less than −15). * Critical checkpoint.
